# Experience of safety monitoring in the context of a prospective observational study of artemether-lumefantrine in rural Tanzania: lessons learned for pharmacovigilance reporting

**DOI:** 10.1186/1475-2875-9-S2-P24

**Published:** 2010-10-20

**Authors:** Abdunoor Mulokozi Kabanywany, Nathan Mulure, Christopher Migoha, Aggrey Malila, Christian Lengeler, Raymond Schlienger, Blaise Genton

**Affiliations:** 1 Ifakara Health Institute, P.O. Box 78373, Kiko Avenue, Old Bagamoyo Road, Mikocheni, Dares Salaam, Tanzania; 2 Novartis Pharma Inc, Nairobi, Kenya; 3 Tanzanian Food and Drugs Authority, Dar es Salaam, Tanzania; 4 Novartis Pharma AG, Basel, Switzerland; 5 Swiss Tropical and Public Health Institute, Basel, Switzerland, Basel, Switzerland

## Objectives

To identify and implement strategies that help meet safety monitoring requirements in the context of an observational study for artemether-lumefantrine (AL) administered as first-line treatment for uncomplicated malaria in rural Tanzania.

## Methods

Pharmacovigilance procedures were developed through collaboration between the investigating bodies (Ifakara Health Institute and Swiss Tropical and Public Health Institute), the relevant regulatory authority (Tanzania Food and Drugs Authority) and the manufacturer of AL (Novartis Pharma AG). Training and refresher sessions on the pharmacovigilance system were provided for healthcare workers from local health facilities and field recorders of the Ifakara Health Demographic Surveillance System (IHDSS). Three distinct channels for identification of adverse events (AEs) and serious adverse events (SAEs) were identified and implemented.

## Results

Training was provided for 40 healthcare providers (with refresher training 18 months later) and for six field recorders. During the period 1^st^ September 2007 to 31^st^ March 2010, 57 AEs were reported (including 43 under AL, four under sulphadoxine-pyrimethamine, one under metakelfin, two after antibiotics). The remaining seven were due to anti-pyretic or anti-parasite medications. Twenty patients experienced SAEs; in 16 cases, a relation to AL was suspected. Six of the 20 cases were reported within 24 hours of occurrence. In all cases reported after AL, 5 cases (vomiting), 16 cases (itching and/or rash) and others were difficult breathing, convulsion and headache (10 cases). The four AEs that occurred after SP were mild erythematic skin lesions that did not progress to Stevens-Johnson syndrome. The AEs seen following treatment with penicillin and amoxicillin were both rashes.

## Discussion

Safety monitoring and reporting is possible even in settings with weak health infrastructure. Reporting can be enhanced by regular and appropriate training of healthcare providers. SMS text alerts provide a practical solution to communication challenges.

## Conclusion

Experience gained in this setting could help to improve spontaneous reporting of AEs and SAEs to health authorities or marketing authorization holders.

